# Ocular Biometry Distribution and One-Year Growth in Eight-Year-Old Southern European Schoolchildren Under the CISViT Project

**DOI:** 10.3390/children12020221

**Published:** 2025-02-12

**Authors:** Mariam El Gharbi, Laura Guisasola, Alba Galdón, Valldeflors Vinuela-Navarro, Joan Pérez-Corral, Núria Tomás, Núria Vila-Vidal

**Affiliations:** 1 Visió Optometria i Salut, Departament d’Òptica i Optometria, Universitat Politècnica de Catalunya, 08222 Terrassa, Spain; laura.guisasola@upc.edu (L.G.); alba.galdon@upc.edu (A.G.); valldeflors.vinuela@upc.edu (V.V.-N.); juan.enrique.perez@upc.edu (J.P.-C.); nuria.tomas@upc.edu (N.T.); nuria.vila@upc.edu (N.V.-V.); 2 Centre Universitari de la Visió, Universitat Politècnica de Catalunya, 08222 Terrassa, Spain

**Keywords:** axial length, corneal radius, ocular biometry, myopia, Southern Europe, schoolchildren

## Abstract

**Objective**: To analyse variations in axial length (AL), corneal radius (CR) and the AL/CR ratio over one year in eight-year-old schoolchildren, considering sex, ethnicity and refractive error. **Methods**: Vision screenings were conducted in 16 schools in Terrassa (Barcelona, Spain) with eight-year-old children as part of the CISViT project. Measurements included ocular biometrics (AL and CR) and non-cycloplegic autorefraction for refractive error. Parental questionnaires provided demographic data (birth date, ethnicity). The same procedures were repeated after one year. **Results**: Ocular biometric parameters differed by sex and ethnicity. Boys and children of Maghreb descent had longer AL and flatter CR than girls and Caucasian children (*p* < 0.001 for both visits). The AL/CR ratio was higher in boys than girls (*p* = 0.002 in the initial visit and *p* = 0.011 in the follow-up visit) but consistent across ethnicities (*p* = 0.291 and *p* = 0.390). AL and AL/CR ratio differed significantly by refractive error status (*p* < 0.001 in both visits), increasing in more myopic children, while CR showed no significant difference. In myopic children, the AL/CR ratio exceeded 3.0, and typical sex-based biometric differences diminished. Growth rates for AL and AL/CR ratio were similar across sex and ethnicity, indicating minimal demographic influence. **Conclusions**: AL and CR differ significantly by sex and ethnicity, with demographic differences evident in baseline measurements but not in growth rates over one year. The consistency of the AL/CR ratio across ethnicities, despite sex-based differences, supports its utility as a reliable metric for assessing refractive development in diverse populations.

## 1. Introduction

Myopia has emerged as a global public health concern in recent decades, significantly affecting a large portion of the population, particularly school-aged children [1,2,3,4,5,6]. Early onset of myopia is linked to the development of high myopia (≥−6 D) in adulthood, which increases the risk of retinal and optic nerve pathologies, potentially leading to severe visual impairment or blindness [1,7]. Myopia and its associated complications are one of the leading causes of visual impairment and blindness worldwide [8,9].

Given the increasing prevalence and potential complications of myopia, it is crucial to have effective methods to identify children at risk of developing or increasing myopia. Ocular biometry measurements, which provide detailed data on axial length (AL) and corneal curvature radius (CR), as well as the ratio between them (AL/CR), are important indicators for the prevention and management of childhood myopia [10,11,12].

Studies have shown that the CR undergoes significant variations during the first three or five years of life and stabilises thereafter [13,14]. In contrast, AL experiences significant growth until adolescence [10,11,12,14]. The myopic changes in refractive error are related to the excessive growth of AL, resulting in a mismatch with CR and other ocular biometric parameters. Therefore, although the myopic changes in refractive error are mainly related to the excessive growth of AL, analysing the AL value alone is not sufficient to identify children at risk of developing myopia; it is necessary to consider both its growth rate and its relationship with CR. The AL/CR ratio can serve as an important indicator of the onset and progression of myopia [12,14].

Longitudinal cohort studies of AL are more important than cross-sectional studies for identifying children at risk of myopia because they allow the monitoring of changes over time within the same individuals, providing a dynamic understanding of eye growth and myopia progression. These studies better predict future refractive changes, evaluate the effectiveness of myopia control treatments, enhance the understanding of the mechanisms behind myopia development and progression and examine correlations between AL and other ocular parameters, offering a comprehensive view that cross-sectional studies cannot capture. This long-term predictive capacity is crucial for pediatric refractive development and supports the creation of practical tools, such as online myopia progression or myopia risk calculators, which can help translate research findings into clinical practice. Such tools bridge the gap between empirical data and real-world applications, aiding clinicians in explaining potential myopia progression to parents and guiding personalized myopia management strategies.

The AL development is influenced by various demographical factors, with age, sex, ethnicity and anthropometry being the most significant [10,14,15,16,17,18]. Geographical region of residence is also a critical variable, as there are notable differences in both the value and growth rates of AL between European and Asian populations, especially in school-aged children [10,12]. While numerous cohort studies have been conducted on Asian and Northern European populations, research in Southern Europe remains sparse. So, considering the impact of environmental and cultural factors on myopia development, studies in diverse populations are crucial.

The Terrassa Visual Health Children’s Cohort (CISViT) project is a pioneering schoolchildren cohort study in the Southern European population. With data collected from the CISViT project, this study aims to analyse variations in AL, CR and the ratio AL/CR over a one-year period in eight-year-old schoolchildren, taking into account sex, ethnicity and refractive error. By doing so, it seeks to provide valuable insights into regional differences in ocular biometric growth patterns and their implications for myopia prevention and management.

## 2. Materials and Methods

### 2.1. Study Design

As part of the CISViT project, which involves 16 schools in the city of Terrassa (Barcelona, Spain), this prospective longitudinal epidemiological cohort study consists of two phases. The initial data collection phase involves a visual screening of eight-year-old schoolchildren at the Centre Universitari de la Visió (University Vision Center; the Universitat Politècnica de Catalunya optometry clinic) comprising monocular measurements of distance visual acuity in both eyes using Snellen charts, non-cycloplegic objective refraction measurements by WAM 5500 open-field autorefractor (Grand Seiko Co., Tokyo, Japan) and ocular biometric measurements, including AL and CR, by MYAH biometer (Topcon Healthcare Co., Tokyo, Japan). Additionally, a distance and near cover test was conducted to detect binocular vision misalignments. In the follow-up phase, a visual screening was conducted one year later within the school premises consisting of monocular non-cycloplegic objective refraction measurements in both eyes (ARK-1e autorefractor, Nidek Co., Gamagori, Japan) and ocular biometric measurements, including AL and CR, by AL-SCAN M biometer (Nidek Co., Japan).

For logistic reasons, participants were screened using different autorefractometers and biometers in the initial and follow-up phases. This should not result in significant differences in outcomes, as a previous study [19] has demonstrated minimal differences between open-field autorefractometers without fogging, such as the WAM 5500, and closed-field autorefractometers with fogging, like the Nidek ARK-1e. In cases of fluctuating measurements or discrepancies with visual acuity, retinoscopy was performed as an additional verification method. On the other hand, a comparative study between the two biometers used in the CISViT project [20] confirmed that they both provide reproducible and interchangeable data, showing a within-subject standard deviation of 0.01 mm and a repeatability limit of 0.04 mm, ensuring consistency and reliability across the study phases. In addition to these tests, before each visual screening, the participants’ parents were required to complete a detailed questionnaire, which collected, among other information, ethnic ancestry and birth data.

### 2.2. Ethics Statement

This study was approved by the Drugs Medication Ethics Committee (CEIm) of Mútua Terrassa (P/22-090) and designed in accordance with the Declaration of Helsinki.

Written informed consent was obtained from each parent or guardian prior to the schoolchildren’s involvement in the study, ensuring that they fully understood the nature and purpose of the research. Informed consent was required and re-confirmed at the follow-up visit.

### 2.3. Study Population Sample

The study involved 476 eight-year-old schoolchildren enrolled in primary schools in Terrassa. A sample size calculation conducted with EpiData software (EpiData Association, Version 4.2) determined that a minimum of 435 participants was needed, assuming a 95% confidence level.

Applying the inclusion criteria of astigmatisms equal to or less than 1.50 D and anisometropia not exceeding 1.50 D, the refractive error of the sample, measured in terms of spherical equivalent (SE), ranged from +3.00 to −4.25 D.

To ensure the validity and reliability of the results, specific exclusion criteria were applied. Children undergoing any myopia control treatments were excluded to avoid confounding effects on ocular development. Additionally, children with amblyopia or strabismus were excluded, as these conditions could interfere with typical eye development. Participants were also excluded if they showed low cooperation during screening or if they did not participate in the follow-up phase of the study.

### 2.4. Measurements and Variable Definitions

- Sociodemographic Variables

The sociodemographic variables considered were sex and ethnicity. The sample was classified by ethnicity into Caucasian, Maghreb and Latin, as these were the most representative, and the category of Other, which included schoolchildren of sub-Saharan African, Asian and biracial descent.

- Ocular Biometric Variables

The biometric variables considered were AL, CR and the AL/CR ratio. The CR was calculated as the average of the two principal corneal radii (Equation (1)). The ratio AL/CR was defined as the quotient of these two variables (Equation (2)).

(1) $$RC=\frac{R1+R2}{2}$$

(2) $$AL/CR\;ratio=\frac{AL}{CR}$$

- Refractive Error

To analyse refractive error, SE was used, defined as Equation (3).

(3) $$SE=Sphere+\frac{Cylindre(-)}{2}$$

The classification of SE was based on refractive status. Hyperopia was defined as SE greater than +0.50 D, emmetropia as SE between +0.50 D and −0.50 D (inclusive) and myopia as SE less than −0.50 D. Regarding SE progression, changes between the initial and follow-up visits were categorized as less than or equal to −0.50 D or more negative than −0.50 D.

### 2.5. Statistical Analysis

The Kolmogorov–Smirnov normality test was employed to assess the normal distribution of variables. The AL and CR parameters followed a normal distribution (*p* > 0.05), while SE and the AL/CR ratio did not follow a normal distribution (*p* < 0.001). Given the high correlation between measurements from both eyes for AL (Pearson’s r: 0.908, *p* < 0.001), CR (Pearson’s r: 0.948, *p* < 0.001), AL/CR ratio (Spearman’s rho: 0.925, *p* < 0.001) and SE (Spearman’s rho: 0.882, *p* < 0.001), only data from the right eye was included for the statistical analysis.

To assess differences among the sample groups, an independent *t*-test or ANOVA was used for variables with a parametric distribution, while the Mann–Whitney U test or Kruskal–Wallis test were applied for non-parametric variables. Post hoc analysis was performed to identify specific group differences. All analyses were carried out using SPSS software (version 29.0.2.0), with statistical significance set at *p* < 0.05.

## 3. Results

### 3.1. Sample Population Characteristics

**Figure 1:**
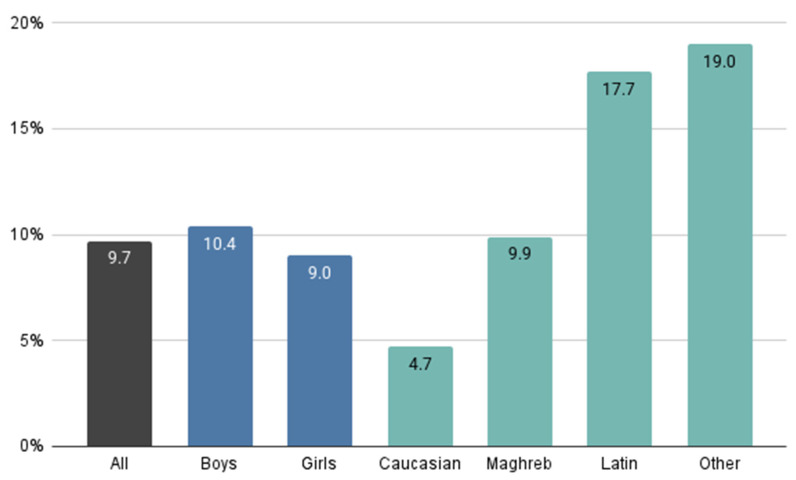
Myopia prevalence at the initial visit, categorised by sex and ethnicity.

This study included 478 schoolchildren who met the inclusion criteria. After excluding two participants due to the inability to measure refractive error during the follow-up visit, the final sample size was 476 schoolchildren. At the initial visit the mean age was 8.2 ± 0.5 years, which increased to 9.3 ± 0.5 years at the one-year follow-up visit. The study sample benefited from a wide ethnic diversity given that it comprised children of Southern European descent as well as a substantial proportion of children of Maghreb descent, a population that has not been previously studied. Table 1 details the population sample demographic characteristics including sex, ethnicity and refractive error, and Figure 1 shows myopia prevalence found for the different demographic characteristics considered at the initial visit.

### 3.2. General Ocular Biometric Parameter Distribution

Table 2 shows the distribution of ocular biometric parameters found at the initial visit and the one-year follow-up. Significant differences were observed between boys and girls across all ocular biometric parameters in both the initial and follow-up visits. Boys exhibited a longer AL (*p* < 0.001 in both visits), a flatter CR (*p* < 0.001 in both visits) and a higher AL/CR ratio (*p* = 0.002 in the initial visit and *p* = 0.011 in the follow-up visit) compared to girls.

### 3.3. Ocular Biometric Parameters by Refractive Error

As shown in Table 3, AL values differed significantly among the refractive groups (*p* < 0.001 in both the initial and follow-up visits). As expected, a greater AL was associated with myopic refractive error. Post hoc analyses revealed significant differences in AL (*p* < 0.001 in both visits) across all three refractive groups. In contrast, no differences in CR were observed across refractive groups (*p* = 0.069 in the initial visit and 0.149 in the follow-up visit).

The refractive groups also exhibited statistically significant differences in the AL/CR ratio (*p* < 0.001 in both visits), with higher ratios in groups with more myopic SE, primarily due to differences in AL. Notably, the myopic group displayed AL/CR ratio values exceeding 3.0 at both visits.

When analysing the distribution of ocular biometry across refractive groups stratified by sex, significant differences were observed in the hyperopic and emmetropic groups only (Table 3). Boys exhibited a longer AL and a flatter CR compared to girls in both visits. At the initial visit, the AL/CR ratio was higher in hyperopic and emmetropic boys compared to girls. However, at the follow-up visit, while this difference persisted in the hyperopic group, no differences were found between emmetropic boys and girls. Conversely, no differences by sex were identified in the myopic group for any biometric parameter at any visit.

### 3.4. Correlation Between Ocular Biometric Parameters and Refractive Error

A significant positive correlation was observed between AL and CR (Pearson coefficient 0.676, *p* < 0.001), indicating that subjects with a longer AL tend to have a flatter CR. As shown in Table 4, SE was inversely correlated with both AL and the AL/CR ratio, suggesting that these variables were higher in individuals with more negative SE. AL was not correlated with SE in hyperopes, and the significant correlation was stronger in myopes than in emmetropes. The correlation coefficient was higher for the AL/CR ratio than for AL alone. No correlation was observed between CR and SE in any of the refractive groups.

### 3.5. Ocular Biometric Parameters by Ethnicity

As shown in Table 5, significant differences were found in AL (*p* = 0.002 in the initial visit and *p* < 0.001 in the follow-up) and CR (*p* = 0.030 in the initial visit and *p* = 0.018 in the follow-up) among the ethnic groups. Post hoc analysis identified that these differences were primarily between Caucasians and Maghreb (*p* < 0.001), with Maghreb displaying a longer AL and flatter CR. In contrast, the AL/CR ratio distribution was similar across ethnic groups (*p* = 0.291 in the initial visit and 0.390 in the follow-up).

Regarding sex, boys had a longer AL (*p* < 0.001 in both visits) than girls across all ethnic groups. Boys also had a significantly flatter CR in most of the ethnic groups, except in Maghreb where the differences in CR between boys and girls is minimal and without statistical significance. Caucasian and Maghreb boys showed higher values in AL/CR ratio than girls; no significant differences in the AL/CR ratio by sex were found in Latin and other ethnicities.

### 3.6. Ocular Biometric Parameters Compared Across Ethnicities

When comparing our results with findings from other populations [6,10] (Table 6), it is observed that the AL in Southern Europe is lower than in Northern Europe (The Netherlands) [10], while the CR is very similar. On the other hand, the results obtained in the Maghreb population closely resembled those in East Asia (China) [6]. Non-differences were observed in the AL/CR ratio between all ethnicities.

### 3.7. Annual Growth in Ocular Biometric Parameters

Table 7 shows the one-year growth of the ocular biometric parameters. Significant growth in AL (*p* = 0.010) and AL/CR ratio (*p* < 0.001) was observed over the follow-up year, while CR remained constant (*p* = 0.574). This suggests that the significant AL/CR ratio variation was primarily due to AL growth.

**Figure 2:**
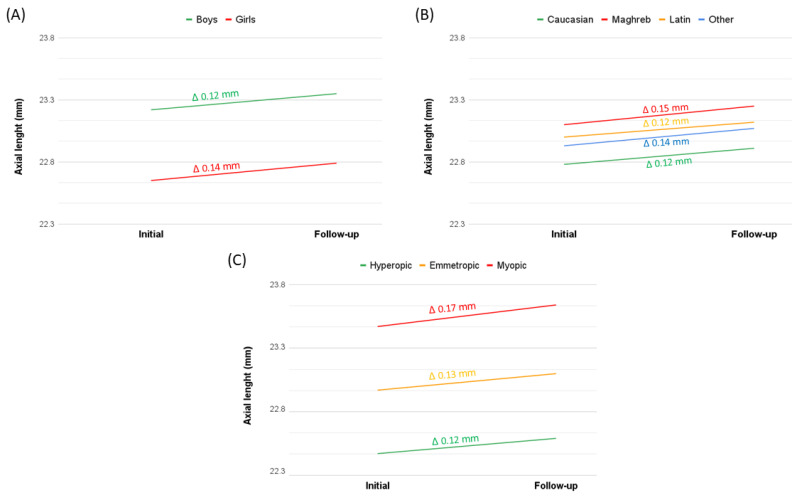
One-year axial length growth (Δ mm) categorised as follows: (**A**) by sex, (**B**) by ethnicity, (**C**) by refractive error groups.

Although no significant sex differences were found in AL growth (*p* = 0.372) (Figure 2A), girls exhibited a statistically significant variation in AL growth (*p* = 0.037), whereas boys did not (*p* = 0.072). No differences by sex were observed in CR (0.296) and AL/CR ratio (*p* = 0.863) variation. Considering ethnicity, there were no differences in the AL growth (*p* = 0.667) (Figure 2B), CR (*p* = 0.436) and the AL/CR ratio (*p* = 0.851) among the different ethnic groups.

When analysing refractive error, significant variation was observed only in the emmetropic group, despite the absence of statistically significant differences in AL (Figure 2C) and AL/CR ratio variations across refractive error groups. Clinically, the myopic group exhibited greater variation but was not statistically significant.

On the other hand, we analysed the variation in SE to identify which groups within the stratified sample—classified by sex, ethnicity and refractive error—showed the greatest variability. As expected, the group showing a SE variation more negative than −0.50 D exhibited greater AL growth (Table 7).

**Figure 3:**
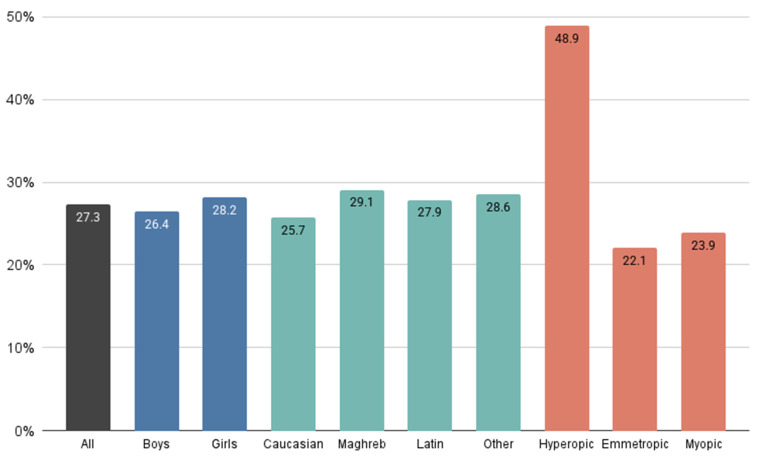
Proportion of schoolchildren with a SE variation more negative than −0.50 D over a one-year follow-up period, categorised by sex, ethnicity and initial refractive error. Refractive error categories were defined based on data from the initial visit.

Figure 3 illustrates the percentage of schoolchildren exhibiting a SE variation more negative than −0.50 D during the one-year follow-up, with data stratified by sex, ethnicity and refractive error. No significant differences were observed in refractive error variations between sexes or ethnicities. However, it is important to emphasize that this negative SE shift was most pronounced among children initially classified as hyperopic, indicating a higher risk of myopia progression in this refractive group compared to emmetropic and myopic children.

In the group with a SE variation more negative than −0.50 D, the SE variation was −0.80 D in hyperopes and −1.01 D in myopes. This trend was reflected in axial length (AL) growth, with hyperopes showing a mean increase of 0.12 mm compared to 0.17 mm in myopes (Table 7 and Figure 2C).

## 4. Discussion

The objective of this study was to provide ocular biometric parameters and monitor their growth over one year in a Southern European population, comparing their influence by sex, ethnicity and refractive error. In addition to comparing the distribution and progression among schoolchildren of diverse ethnic backgrounds from the same geographic region, the study also compared the results with those from studies conducted in Norther Europe and East Asia.

### 4.1. Ocular Biometric Parameter Distribution by Sociodemographic Variable

Significant sex-based differences were found in ocular biometric parameters, with girls showing a shorter AL, a steeper CR and a lower AL/CR ratio. These findings were consistent with previous studies in both European [10,14,18,21,22] and Asian populations [5,11,12,17,23,24]. This may be related to the strong correlation between AL and both height and BMI reported in these studies [5,10,11,18,24]. It has been documented that in emmetropic individuals, body growth and axial elongation are correlated, whereas in myopic individuals, body growth appears to stabilize while axial elongation continues at an accelerated rate, suggesting a dysregulation of normal ocular growth [5,18]. Our findings align with this observation, as sex-based differences lose significance in the presence of myopia. However, further research is needed to better understand the relationship between ocular and body parameters.

Significant differences in the distribution of ocular biometric parameters were also observed across ethnic groups, particularly between children of Caucasian and Maghreb descent. Children of Maghreb descent exhibited a longer AL and a flatter CR, although the AL/CR ratio was similar between the two groups. Similar differences were observed when comparing the ethnic groups in our study with populations from Northern Europe [10] and East Asia [6]. This highlights that differences in ocular biometric parameters exist not only between geographic regions but also among children born and raised in the same country, who were exposed to similar environmental factors yet differed in ethnic origins, as was the case in our sample.

Harrington et al. [25] similarly reported that, within the same region, individuals of European descent have shorter ALs compared to those of non-European descent, which is consistent with our findings. These results suggest that genetic factors play a significant role in determining ocular dimensions, even in the presence of shared environmental influences.

This study revealed a relationship between AL and CR, where eyes with greater AL are generally associated with a flatter CR. This finding may help explain why ethnic groups with a longer AL, as well as boys compared with girls, typically show a significantly flatter CR. These observations are consistent with previous evidence [11,12,14,17,21,22,23], showing that males generally exhibit a flatter CR than females across most of the ethnicities studied.

Interestingly, however, no differences were found in the CR between Maghreb boys and girls, even significant differences were found in the AL and AL/CR ratio. To date, no scientific evidence regarding ocular biometric parameters in the Maghreb population has been documented, making this study the first to analyse these characteristics and providing new insights into this population.

The differences in AL and CR across ethnicities result in a similar AL/CR ratio across the ethnicities included in this study and the studies from Northern Europe [10] and East Asia [6], suggesting that the AL/CR ratio is the only biometric variable that does not differ across ethnicities.

### 4.2. Ocular Biometric Parameters by Refractive Error

The AL/CR ratio has been investigated as a method to analyse the relationship between these two parameters in relation to refractive error development. Previous studies have established that the CR, along with the AL and the lens thickness, is one of the most important determinants of refraction [11], though its role in myopia progression is less relevant [14,26]. Our findings are consistent with these studies, as no significant variations in CR were observed at this age. Consequently, variations in the AL/CR ratio are linked to the AL growth.

Our analysis confirms that the correlation between the AL/CR ratio and SE is stronger than the correlation between the AL and SE alone, particularly as SE becomes more negative. Notably, our findings validate an AL/CR ratio of 3.0 as a diagnostic cutoff value for myopia, in alignment with prior studies in both North-European and Asian populations. He et al. [27] and Tideman et al. [10] reported a stronger correlation between SE and both AL and AL/CR ratio in myopic eyes compared to emmetropic ones. Ip et al. [28] found that although a longer AL is associated with higher likelihood of myopia, cases of myopia were also observed in eyes with shorter AL, indicating that the AL/CR ratio may be a more reliable indicator of myopia risk than AL alone [6,10,12,14]. Liu et al. [29] reported that the AL/CR ratio provided the best prediction of myopia using age-dependent cutoff values for all but preschool children.

This study reveals that the differences in AL and CR between boys and girls lose significance in the myopic group. This could be due to axial elongation becoming the dominant factor driving changes in eye structure, potentially masking the typical sex-related differences observed in emmetropic or hyperopic children. Multiple studies have shown that AL growth is similar between boys and girls, while CR remains stable with age [10,12,22,30]. This pattern suggests that the accelerated growth of myopic eyes may reduce sex-based differences.

Furthermore, these compensatory differences in AL and CR result in minimal differences in myopia prevalence based on sex. Tideman et al. [10] reported minimal SE differences between boys and girls at both nine years old and in adulthood, which aligns with our analysis, where sex-based differences in AL and CR result in non-significant difference in refractive error prevalence. NICER [30], WEPRoM [2] and STEM [3] studies also revealed that there was no significant sex difference in the prevalence of myopia or hyperopia in pre-adolescent children. However, there were several studies that found that girls had significantly higher myopia prevalence than boys [11,12,31].

### 4.3. Annual Growth in Ocular Biometric Parameters

The annual AL growth rate found in our study, 0.13 mm/year, is lower than rates reported in North European cohort studies. Tideman et al. [10] found a growth rate of 0.21 mm/year in children aged 6 to 9, although it is important to note that this value was calculated by averaging the rate over a three-year period. In the German LIFE cohort study [21], AL growth was 0.30 mm/year in three-year-old children, decreasing to 0.10 mm/year from the age of 13 onwards. The growth rate in our study is similar to that reported by the NICER study [32], which observed a growth of 0.12 mm/year at the 50th percentile for children aged 6 to 16 years, although follow-up visits in this study were not annual but conducted every three years.

In our analysis, AL growth in girls was statistically significant, while in boys it was not; however, there were no significant differences in the AL growth rate between sexes. The LIFE study [22] reported that AL growth rates are similar in boys and girls until age 10, after this age, the AL in girls continues to grow until around age 14, while no significant growth occurs in boys. The Shanghai cohort study [24] similarly observed no sex-based differences in children aged 6 to 8 years, suggesting that sex may not be a predictive factor of accelerated AL growth.

Regarding ethnicity, we found that the annual growth rate was similar across different ethnic groups. This could be related to the fact that all participants in the sample live in the same city and share a similar educational environment, meaning that visual demands and environmental factors are comparable. Maybe if these populations were analysed in their countries of origin, different growth patterns would be observed.

Given the observed distribution of AL and CR by sex and ethnicity, along with the similar growth rates, we can suggest that genetic factors primarily influence baseline ocular dimensions, while environmental factors may play a more significant role in growth dynamics.

Regarding CR, previous studies have reported minimal changes in CR throughout different stages of growth [10,11,14]. Our findings align with these studies, as CR showed no significant variation over the one-year follow-up. These minimal variations in CR indicate that its impact on negative SE changes is limited at this stage of development [14]. For this reason, we did not find a significant correlation between CR and SE in our analysis, indicating that the variable responsible for the growth of the AL/CR ratio and, therefore, myopic SE progression is the AL growth.

Regarding the AL/CR ratio, the annual increase rate was 0.021, consistent across sexes and ethnicities. This aligns with the annual rate of 0.025 in European children aged 6 to 9 years reported by Tideman et al. [10] and of 0.02–0.04 in Chinese children aged 6 to 12 years reported by He et al. [12].

While most studies have examined the AL/CR ratio as a predictor of myopia onset, there is limited evidence regarding its usefulness as a predictor of myopia progression. The longitudinal COMET study [14] found a non-significant relationship between the AL/CR ratio and myopia progression. This may explain the non-significant differences in AL/CR ratio growth in groups with differing SE changes. However, we cannot draw reliable conclusions from a one-year follow-up analysis; a longer follow-up period is needed.

On the other hand, as shown in Figure 3, schoolchildren classified as hyperopic during the initial visit exhibited a higher percentage of SE changes more negative than −0.50 D, while the percentage of schoolchildren with SE variation more negative than −0.50 D was similar between myopes and emmetropes. Although the SE variation and AL growth were greater in myopic schoolchildren compared to hyperopic ones, the hyperopic population demonstrated greater variability. This observation may suggest that hyperopic children have a greater predisposition to myopic changes. Future follow-up assessments will be necessary to determine whether the proportion of hyperopic children who demonstrated this negative SE variation will ultimately progress to myopia. Therefore, we recommend that, during clinical practice, close attention be given to SE variations and AL growth in the hyperopic children, similar to the attention typically given to myopic children.

In summary, our study provides valuable insights into the distribution and growth of ocular biometric parameters in Southern Europe by sex and ethnic groups. This is the first study to analyse these parameters in a Southern European population with significant ethnic representation, offering a unique perspective on ocular development in the region. Although sex-based differences in AL and CR exist, their impact on refractive errors remains minimal due to compensatory mechanisms, as seen in previous studies. Notably, we found that the hyperopic group exhibited the greatest variation in SE. Therefore, changes in SE and AL should be carefully monitored regardless of the refractive group to which children belong, as they may have a predisposition to developing myopia. The similarities in AL growth rates among different ethnic groups in our sample suggest that environmental factors, such as shared living and educational environments, may play a more significant role in growth patterns than in the baseline ocular dimensions. However, the limited follow-up period of one year restricts the ability to draw definitive conclusions about the progression of these changes over time, emphasising the need for longer longitudinal studies to fully understand the dynamics of ocular development in diverse populations.

## 5. Strengths and Limitations

This is the first longitudinal study analysing ocular biometric parameters conducted using a Southern European population with a substantial sample size. Considering the reported differences between ethnic groups, longitudinal studies of this population are necessary to determine the variations that occur in participants, providing a long-term perspective on evolution during growth. Additionally, the study offers an epidemiological perspective, representing the demographic and refractive diversity of the population studied. Annual follow-up was conducted, making this one of the few studies to analyse growth over short periods of time, thereby capturing small variations.

However, the study has some limitations. The study employs a rigorous protocol with accommodation controlled through an open-field auto refractometer in the initial phase and, in cases where results fluctuated or showed discrepancies with visual acuity, retinoscopy was used in both the initial and follow-up phases, but cycloplegic refraction was not used. While cycloplegic refraction is the gold standard, several studies analysing biometric parameters use non-cycloplegic measurements. Additionally, various epidemiological studies do not use cycloplegia, such as the WEPrOM Study [2], which defends that the non-cycloplegic SE can facilitate risk profiling of children at risk of myopia onset under pragmatic settings. Furthermore, in our study, refraction was only used as a categorical variable, further reducing potential measurement errors due to the absence of cycloplegia.

Another limitation involves the use of different equipment for measurements during the two study phases. However, comparative analyses demonstrate that the results remain consistent, ensuring the robustness of findings across phases.

## 6. Future Research Directions

The CISViT project aims to extend the follow-up period to three years. This will allow for a more comprehensive and accurate assessment of ocular development progression, providing a better understanding of long-term changes in AL and the myopia progression. Additionally, the study aims to analyse other variables such as height, weight and body mass index, as these may influence ocular growth.

## Figures and Tables

**Table 1:** Distribution of refractive error groups and ethnicity in the study sample at the initial visit, categorised by sex. *p*-values indicate differences by sex.

	All N = 476	Boys N = 231 (48.5%)	Girls N = 245 (51.5%)	*p*
**Refractive Error**				
Hyperopic	90 (18.9%)	43 (18.6%)	47 (19.2%)	0.673
Emmetropic	340 (71.4%)	164 (71.0%)	176 (71.8%)	0.515
Myopic	46 (9.7%)	24 (10.4%)	22 (9.0%)	0.768
**Ethnicity**				
Caucasian	214 (45%)	106 (45.9%)	108 (44.1%)	0.891
Maghreb	141 (29.6%)	69 (29.9%)	72 (29.4%)	0.801
Latin	79 (16.6%)	38 (16.5%)	41 (16.7%)	0.736
Other	42 (8.8%)	18 (7.8)	24 (9.8%)	0.355

**Table 2:** Ocular biometric parameter distribution categorised by sex at the initial and the one-year follow-up visits. *p*-values indicate differences between boys and girls.

	Initial Visit (8.2 Years Old)				Follow-Up Visit (9.3 Years Old)			
	All	Boys	Girls	*p*	All	Boys	Girls	*p*
AL (mm)	22.93 ± 0.78	23.22 ± 0.73	22.65 ± 0.72	**<0.001**	23.06 ± 0.81	23.35 ± 0.75	22.79 ± 0.77	**<0.001**
CR (mm)	7.79 ± 0.27	7.86 ± 0.27	7.72 ± 0.25	**<0.001**	7.78 ± 0.27	7.85 ± 0.28	7.71 ± 0.25	**<0.001**
AL/CR	2.95 ± 0.08	2.96 ± 0.09	2.93 ± 0.08	**0.002**	2.97 ± 0.09	2.98 ± 0.09	2.96 ± 0.08	**0.011**

**Table 3:** Ocular biometric parameter distribution categorised by sex and refractive error at both the initial and one-year follow-up visits. *p* (sex) indicates differences between boys and girls and *p* (refractive error) indicates differences among refractive error groups.

	Initial Visit (8.2 Years Old)					Follow-Up Visit (9.3 Years Old)				
	All	Boys	Girls	*p* (Sex)	*p* (Refractive Error)	All	Boys	Girls	*p* (Sex)	*p* (Refractive Error)
**AL (mm)**					**<0.001**					**<0.001**
All	22.93 ± 0.78	23.22 ± 0.73	22.65 ± 0.72	**<0.001**		23.06 ± 0.81	23.35 ± 0.75	22.79 ± 0.77	**<0.001**	
Hyperopic	22.47 ± 0.68	22.75 ± 0.65	22.21 ± 0.60	**<0.001**		22.59 ± 0.68	22.88 ± 0.65	22.32 ± 0.61	**<0.001**	
Emmetropic	22.97 ± 0.74	23.29 ± 0.69	22.68 ± 0.67	**<0.001**		23.10 ± 0.76	23.40 ± 0.70	22.82 ± 070	**<0.001**	
Myopic	23.47 ± 0.68	23.62 ± 0.76	23.29 ± 0.83	0.169		23.64 ± 0.92	23.77 ± 0.85	23.50 ± 0.99	0.332	
**CR (mm)**					0.069					0.149
All	7.79 ± 0.27	7.86 ± 0.27	7.72 ± 0.25	**<0.001**		7.78 ± 0.27	7.85 ± 0.28	7.71 ± 0.25	**<0.001**	
Hyperopic	7.75 ± 0.23	7.81 ± 0.24	7.69 ± 0.22	**0.0017**		7.74 ± 0.23	7.80 ± 0.24	7.69 ± 0.22	**0.022**	
Emmetropic	7.81 ± 0.28	7.88 ± 0.28	7.73 ± 0.26	**<0.001**		7.79 ± 0.29	7.88 ± 0.29	7.71 ± 0.26	**<0.001**	
Myopic	7.73 ± 0.23	7.76 ± 0.19	7.71 ± 0.27	0.512		7.73 ± 0.23	7.74 ± 0.20	7.71 ± 0.27	0.670	
**AL/CR**					**<0.001**					**<0.001**
All	2.95 ± 0.08	2.96 ± 0.09	2.93 ± 0.08	**0.002**		2.97 ± 0.09	2.98 ± 0.09	2.96 ± 0.08	**0.011**	
Hyperopic	2.90 ± 0.07	2.92 ± 0.07	2.89 ± 0.06	**0.044**		2.92 ± 0.07	2.93 ± 0.07	2.90 ± 0.06	**0.016**	
Emmetropic	2.94 ± 0.07	2.96 ± 0.08	2.93 ± 0.07	**0.024**		2.97 ± 0.07	2.97 ± 0.07	2.96 ± 0.07	0.127	
Myopic	3.04 ± 0.10	3.05 ± 0.11	3.02 ± 0.10	0.612		3.06 ± 0.12	3.07 ± 0.12	3.05 ± 0.11	0.660	

**Table 4:** Spearman’s rho correlation coefficient (*p*-value) for ocular biometric parameters and SE at the initial and follow-up visits.

	Initial Visit (8.2 Years Old)			Follow-Up Visit (9.3 Years Old)		
	AL	CR	AL/CR	AL	CR	AL/CR
All	−0.396 (***p* < 0.001**)	−0.018 (*p* = 0.694)	−0.410 (***p* < 0.001**)	−0.312 (***p* < 0.001**)	0.072 (*p* = 0.117)	−0.413 (***p* < 0.001**)
Hyperopic	−0.204 (*p* = 0.054)	0.104 (*p* = 0.330)	−0.384 (***p* < 0.001**)	−0.014 (*p* = 0.893)	0.196 (*p* = 0.064)	−0.301 (***p* = 0.004**)
Emmetropic	−0.234 (***p* < 0.001**)	−0.011 (*p* = 0.840)	−0.233 (***p* < 0.001**)	−0.229 (***p* < 0.001**)	0.037 (*p* = 0.501)	−0.289 (***p* < 0.001**)
Myopic	−0.603 (***p* < 0.001**)	0.081 (*p* = 0.593)	−0.682 (***p* < 0.001**)	−0.661 (***p* < 0.001**)	0.137 (*p* = 0.365)	−0.767 (***p* < 0.001**)

**Table 5:** Ocular biometric parameter distribution categorised by sex and ethnicity at the initial and one-year follow-up visits. *p* (sex) values indicate differences between boys and girls and *p* (ethnicity) indicate the differences among ethnic groups.

	Initial Visit (8.2 Years Old)					Follow-Up Visit (9.3 Years Old)				
	All	Boys	Girls	*p* (Sex)	*p* (Ethnicity)	All	Boys	Girls	*p* (Sex)	*p* (Ethnicity)
**AL (mm)**					**0.002**					**<0.001**
Caucasian	22.78 ± 0.76	23.09 ± 0.69	22.48 ± 0.70	**<0.001**		22.91 ± 0.78	23.20 ± 0.70	22.61 ± 0.73	**<0.001**	
Maghreb	23.10 ± 0.81	23.38 ± 0.79	22.84 ± 0.74	**<0.001**		23.25 ± 0.86	23.52 ± 0.82	23.00 ± 0.82	**<0.001**	
Latin	23.00 ± 0.74	23.24 ± 0.73	22.77 ± 0.68	**0.004**		23.12 ± 0.76	23.36 ± 0.76	22.91 ± 0.67	**0.008**	
Other	22.93 ± 0.78	23.37 ± 0.57	22.60 ± 0.70	**<0.001**		23.07 ± 0.75	23.53 ± 0.53	22.73 ± 0.72	**<0.001**	
**CR (mm)**					**0.030**					**0.018**
Caucasian	7.75 ± 0.27	7.83 ± 0.28	7.68 ± 0.24	**<0.001**		7.74 ± 0.27	7.82 ± 0.28	7.66 ± 0.24	**<0.001**	
Maghreb	7.84 ± 0.28	7.89 ± 0.28	7.80 ± 0.26	0.057		7.84 ± 0.28	7.88 ± 0.28	7.79 ± 0.26	0.060	
Latin	7.78 ± 0.25	7.85 ± 0.25	7.72 ± 0.68	**0.020**		7.77 ± 0.26	7.84 ± 0.26	7.71 ± 0.25	**0.028**	
Other	7.79 ± 0.27	7.93 ± 0.19	7.70 ± 0.25	**0.002**		7.78 ± 0.27	7.92 ± 0.21	7.67 ± 0.25	**0.001**	
**AL/CR**					0.291					0.390
Caucasian	2.94 ± 0.07	2.95 ± 0.08	2.93 ± 0.06	0.062		2.96 ± 0.08	2.97 ± 0.08	2.95 ± 0.07	**0.025**	
Maghreb	2.95 ± 0.09	2.97 ± 0.09	2.93 ± 0.09	**0.029**		2.97 ± 0.10	2.99 ± 0.10	2.95 ± 0.10	**0.031**	
Latin	2.96 ± 0.09	2.96 ± 0.09	2.95 ± 0.09	0.575		2.98 ± 0.09	2.98 ± 0.10	2.97 ± 0.09	0.623	
Other	2.94 ± 0.07	2.95 ± 0.06	2.94 ± 0.068	0.550		2.97 ± 0.08	2.97 ± 0.08	2.97 ± 0.07	0.848	

**Table 6:** Comparison of ocular biometric parameters and refractive error among nine-year-old schoolchildren across different ethnic origins and studies: Southern European (CISViT study), Northern European (Generation R study) [10], Maghreb (CISViT study) and East Asian (Baoshan Eye study) [6]. The SE was obtained via cycloplegic autorefraction in the Generation R and Baoshan Eye studies.

Population Group	AL (mm)	CR (mm)	AL/CR Ratio	SE (D)	Study
Southern Europe	22.91 ± 0.78	7.74 ± 0.27	2.96 ± 0.08	+0.09 ± 0.78	CISViT
Northern Europe	23.10 ± 0.84	7.78 ± 0.26	2.97 ± 0.09	+0.74 ± 1.30	Generation R [10]
Maghreb	23.25 ± 0.86	7.84 ± 0.28	2.97 ± 0.10	−0.39 ± 1.12	CISViT
East Asia	23.29 ± 0.87	7.84 ± 0.25	2.97 ± 0.10	0.02 ± 1.29	Baoshan Eye [6]

**Table 7:** One-year variation in AL and AL/CR ratio of the sample categorised by sex, refractive error and ethnicity. *p* (variation) indicates significance in the one-year variation and *p* (groups) indicates differences among sexes, refractive error and ethnicity.

	AL (mm)			AL/CR		
	Variation	*p* (Variation)	*p* (Group)	Variation	*p* (Variation)	*p* (Group)
**All**	0.13	**0.010**		0.021	**<0.001**	
**Sex**			0.372			0.863
Boys	0.12	0.072		0.019	**0.021**	
Girls	0.14	**0.037**		0.023	**0.002**	
**Refractive error**			0.484			0.332
Hyperopic	0.12	0.223		0.016	0.116	
Emmetropic	0.13	**0.024**		0.022	**<0.001**	
Myopic	0.17	0.336		0.024	0.312	
**SE variation**			**<0.001**			**<0.001**
∆SE ≥ −0.50 D	0.12	**0.034**		0.020	**0.001**	
∆SE < −0.50 D	0.17	0.144		0.024	**0.038**	
**Ethnicity**			0.667			0.851
Caucasian	0.12	0.110		0.021	**0.004**	
Maghreb	0.15	0.135		0.021	0.071	
Latin	0.13	0.286		0.019	0.181	
Other	0.14	0.390		0.024	0.127	

## Data Availability

The data presented in this study are available upon request from the corresponding author due to ethical reasons.

## Abbreviations

The following abbreviations are used in this manuscript:

AL	Axial length
CISViT	Terrassa Visual Health Children’s Cohort
CR	Corneal radius
D	Diopters
mm	millimeters
SE	Spherical equivalent
∆	Variation
